# Acute exacerbation of anti-Ha antibody-positive antisynthetase syndrome-associated interstitial lung disease: a case report

**DOI:** 10.3389/fimmu.2025.1614777

**Published:** 2025-06-12

**Authors:** Yinying Li, Junyi Ke, Shu Huang, Shiya Lin, Siyu Lei, Hongchun Huang, Luxi Lei, Qinglan Li, Yuanlan Wu, Minchao Duan

**Affiliations:** ^1^ Guangxi Medical University, Nanning, Guangxi, China; ^2^ Department of Respiratory and Critical Care Medicine, Wuming Hospital of Guangxi Medical University, Nanning, Guangxi, China

**Keywords:** anti-synthetase syndrome (ASS), anti-Ha antibody, ILD, case report, corticosteroids therapy

## Abstract

**Background:**

Anti-synthetase syndrome (ASS) is a rare autoimmune myopathy, frequently associated with interstitial lung disease, and is characterized by the presence of anti-aminoacyl tRNA synthetase (ARS) antibodies. However, there have been limited reports of cases exhibiting positive anti-Ha antibodies.

**Methods:**

This study presents a retrospective analysis of the clinical data from a patient with an acute exacerbation of ASS who tested positive for anti-Ha antibodies.

**Case presentation:**

This patient initially presented with interstitial pneumonia. The initial anti-infective treatment was ineffective; however, symptoms improved following the addition of corticosteroids. Upon discontinuation of corticosteroids, the patient experienced a recurrence of cough, progressive worsening of dyspnea, and developed lower general weakness. Comprehensive autoantibody testing revealed positivity for anti-Ha antibodies, and MRI of the lower limbs indicated soft tissue edema. The patient was ultimately diagnosed with ASS with interstitial lung disease. Treatment with methylprednisolone pulse therapy, combined with cyclophosphamide, tacrolimus, tofacitinib citrate, and pirfenidone, led to an improvement in the patient’s condition, resulting in discharge. Post-discharge, the patient was maintained on regular oral prednisone, nintedanib, and tofacitinib. Follow-up to date has shown a stable condition, with resolution of pulmonary lesions observed upon re-examination.

**Conclusion:**

Anti-Ha antibody is one of the specific antibodies associated with ASS, yet its positive rate remains exceedingly low. This case represents the first reported instance of an anti-Ha antibody-positive ASS in China. Misdiagnosis and missed diagnosis are prevalent in clinical practice, underscoring the importance of screening for autoantibodies when patients present with acute, unexplained interstitial lung changes and a poor response to anti-infective treatment. Furthermore, interstitial lung disease is the most common extra-muscular clinical manifestation observed in ASS patients. Differentiating between acute exacerbations of pulmonary infections and interstitial lung disease associated with rheumatic diseases poses a significant challenge, as both can occur concurrently. Therefore, during diagnosis and treatment, it is crucial to consider not only infections but also to identify the underlying causes of worsening lung lesions.

## Introduction

**Figure 1:**
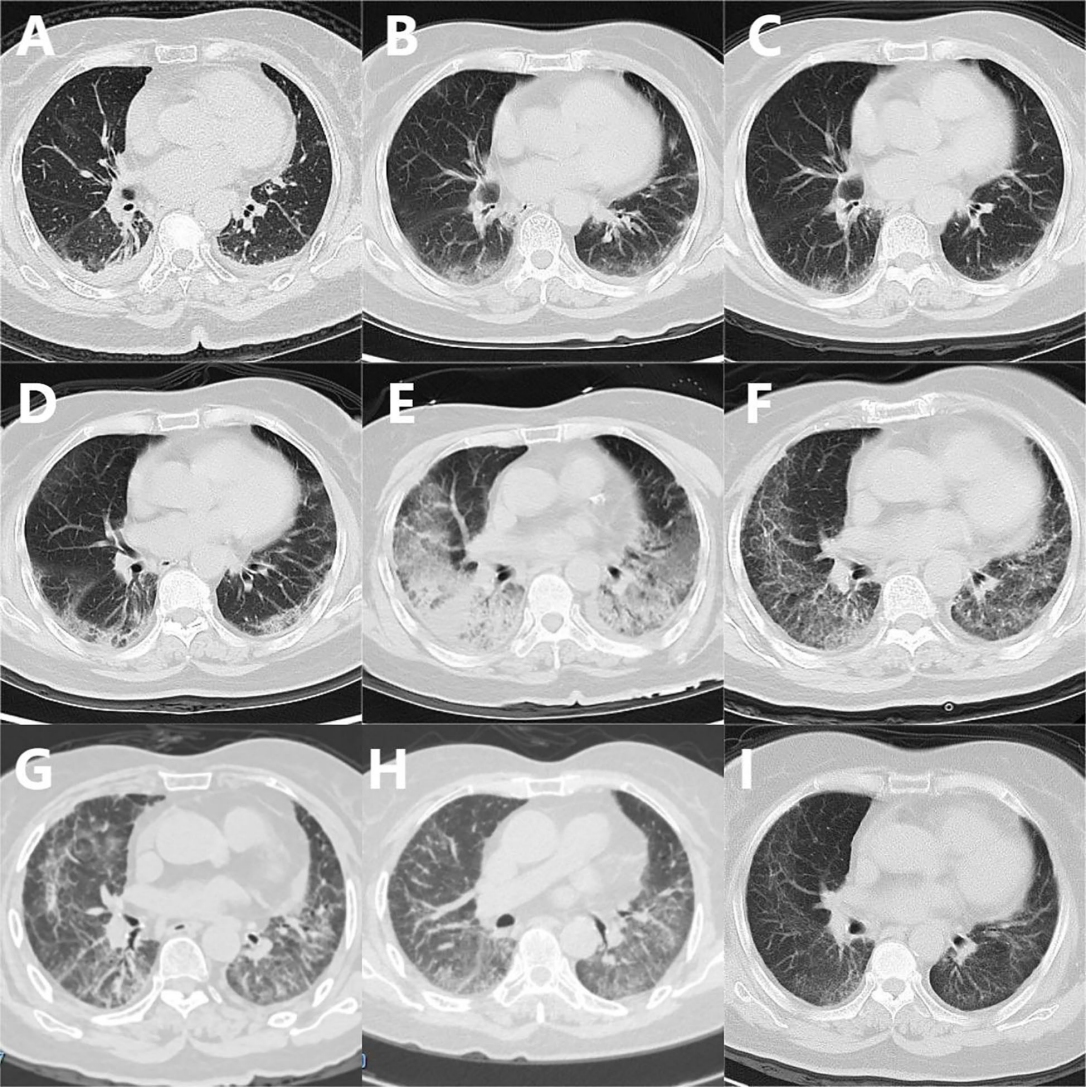
Chest CT of the Patient. **(A)** 2023-01-04: Patchy and linear high-density shadows were observed in both lungs, particularly prominent in the subpleural areas and lower lobes, accompanied by arc-shaped effusion shadows in both pleural cavities. **(B)** 2023-01-14: Ground-glass opacities were noted in both lungs, also prominent in the subpleural areas, alongside scattered patchy and linear high-density shadows with blurred edges, and the disappearance of arc-shaped effusion shadows in both pleural cavities. **(C)** 2023-01-19: A reduction in ground-glass opacities and patchy, linear high-density shadows in the lower lobes of both lungs was observed compared to previous findings. **(D)** 2023-01-27: An increase in ground-glass opacities and patchy, linear high-density shadows in the lower lobes of both lungs was noted compared to prior findings. **(E)** 2023-02-12: Following combined anti-infective and pirfenidone anti-fibrotic therapy. **(F)** 2023-03-25: After the second methylprednisolone pulse therapy and sequential treatment, during which prednisone acetate, tacrolimus, tofacitinib citrate, and pirfenidone were administered for maintenance therapy. **(G)** 2023-06-06: Following anti-infective, immunosuppressive, and anti-pulmonary fibrosis therapy, a reduction in ground-glass opacities and absorption of patchy, linear high-density shadows in both lungs was observed. **(H)** 2023-07-07: Further absorption of ground-glass opacities and patchy, linear high-density shadows in both lungs was noted, with increased lung translucency. **(I)** 2023-11-21: Further reduction of subpleural patchy, linear high-density shadows and ground-glass opacities in both lungs was observed, accompanied by an increase in lung translucency.

The patient, a 69-year-old female, was admitted to the hospital due to a cough persisting for over half a month. She began experiencing paroxysmal and continuous coughing, predominantly at night, on January 1, 2023. There were no symptoms such as fever, chest tightness, shortness of breath, palpitations, sore throat, muscle aches, nasal congestion, or runny nose, and no treatment was administered. A chest Computed Tomography (CT) performed on January 4 revealed significant patchy and cord-like high-density shadows in the subpleural areas and lower lobes of both lungs, along with a small amount of ground-glass opacity. Shallow, arc-shaped water-density shadows were observed in both pleural cavities (**Figure 1A**). Community-acquired pneumonia was considered, and empirical anti-infective treatment with cefixime was initiated for 10 days; however, the cough did not improve. She was subsequently admitted to the hospital on January 14.

**Figure 2:**
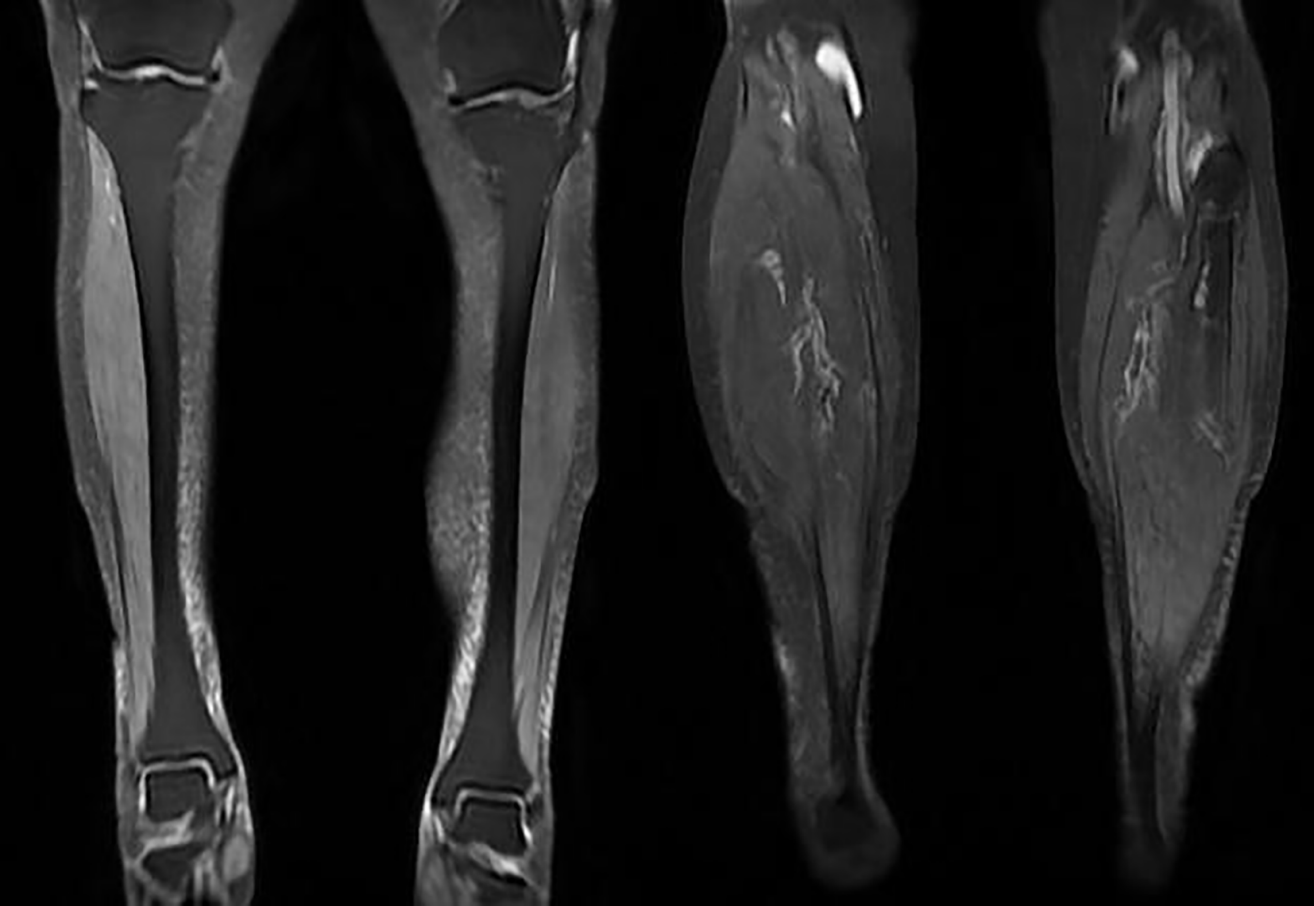
The timeline of treatment process in this case.

Upon admission, the patient’s body temperature was recorded at 36.8°C, with a heart rate of 104 beats per minute and a respiratory rate of 21 breaths per minute. The blood pressure was measured at 110/71 mmHg. There is no cyanosis of the lips, no observed skin rashes, and no palpable superficial lymphadenopathy. Bilateral lung auscultation reveals coarse breath sounds accompanied by a few Velcro rales in the lower lung fields, with no dry rales or pleural friction rub detected. Cardiac and abdominal examinations reveal no significant abnormalities. There is an absence of finger clubbing and no edema present in the lower extremities. Muscle strength and tone in the limbs are assessed as normal.After admission, relevant examinations were conducted. Blood gas analysis revealed a pH of 7.44, arterial PaCO2 of 36 mmHg, arterial PaO2 of 125 mmHg, and an oxygenation index (PO2/FiO2) of 431 mmHg. High-sensitivity C-reactive protein (hsCRP) was measured at 37.28 mg/L, while procalcitonin was at 0.08 ng/ml. The respiratory pathogen panel, which included eleven items, tested positive for Mycoplasma pneumoniae-IgM. Lymphocyte subset analysis showed CD3+ at 708/µl, CD4+ at 516/µl, and CD8+ at 160/µl. Routine blood and urine tests, along with assessments of liver and kidney function, electrolytes, myocardial enzymes, coagulation, BNP, and serum ferritin, revealed no significant abnormalities. Chest CT indicated an increase in ground-glass opacities in both lungs compared to previous findings, predominantly subpleural, with scattered patchy and linear high-density shadows that had blurred margins. The arc-shaped effusion shadows in both pleural cavities had disappeared (**Figure 1B**). Considering the patient’s community-acquired pneumonia and the increased ground-glass opacities in both lungs, empirical treatment with cefoperazone-tazobactam sodium was initiated for anti-infection. On January 16, methylprednisolone at 40 mg/d was added for anti-inflammatory treatment. After four days, the patient’s cough symptoms significantly improved. A follow-up chest CT indicated absorption of the ground-glass opacities and high-density shadows compared to previous findings (**Figure 1C**). On January 20, a re-examination revealed a hsCRP level of 2.88 mg/L, and no abnormalities in the routine blood test. The patient was discharged with advice to take moxifloxacin orally for one week. Occasional coughing persisted post-discharge. On January 26, the patient experienced an exacerbation of cough accompanied by chest tightness and dyspnea, but without fever, chills, nasal congestion, runny nose, sore throat, muscle aches, fatigue, orthopnea, or frothy pink sputum. The patient was subsequently readmitted to our department. Blood gas analysis revealed the following: pH 7.45, arterial PaCO2 32 mmHg, arterial PaO2 54 mmHg, and PO2/FiO2 of 190 mmHg. The complete blood count results indicated white blood cells at 6×10^9/L, neutrophils at 78.4%. hsCRP was measured at 78.62 mg/L; serum ferritin was found to be 1108.85 ng/mL; and Mycoplasma pneumoniae-IgM was weakly positive. Lymphocyte subset analysis showed the following: CD3+ at 399 cells/µL, CD4+ at 231 cells/µL, CD8+ at 131 cells/µL, and CD19+ at 58 cells/µL. Tests for influenza A virus RNA, influenza B virus RNA, SARS-CoV-2 nucleic acid, serum Aspergillus galactomannan, serum Aspergillus IgG antibody, and anti-streptolysin O antibody were all negative.Chest CT revealed a slight increase in ground-glass opacities and linear high-density shadows in the lower lobes of both lungs compared to previous findings, with pleural effusion having been absorbed (**Figure 1D**). Interstitial pneumonia (viral etiology suspected) was considered, and the patient was treated successively with levofloxacin, piperacillin sodium tazobactam sodium, meropenem combined with cotrimoxazole and doxycycline for anti-infection, along with pirfenidone for anti-fibrosis. However, the patient’s respiratory symptoms showed no significant improvement, and systemic fatigue developed. A follow-up chest CT on February 12 indicated a further increase in ground-glass opacities and high-density shadows in the lower lobes of both lungs (**Figure 1E**). Given the patient’s progressively worsening respiratory symptoms, including cough and shortness of breath, unexplained interstitial lung changes, and general fatigue, the possibility of connective tissue disease affecting the lungs cannot be excluded. The treatment plan was adjusted to include: Methylprednisolone 160 mg for 6 days, Human Immunoglobulin 25 g/day, Baricitinib 4 mg/day, and Tacrolimus 2 mg twice daily. Relevant tests were also completed: Rheumatoid factor, five immune molecules, Direct Antiglobulin Test (Coombs), and Antinuclear Antibody Profile showed no abnormalities. On February 17, serum was sent to Wuhan Kangsheng Zhenyuan Medical Laboratory Co., Ltd. (Wuhan, China) for a comprehensive dermatomyositis test, which revealed that Anti-Ha antibody IgG level of 268.07 U/ml, anti-MDA5 antibody IgG level of 7.60 U/ml. Considering the case of Anti-synthetase syndrome (ASS) with positive anti-Ha antibodies, the first and second courses of corticosteroids pulse therapy were administered, consisting of methylprednisolone at a dosage of 120 mg over 2 days, combined with cyclophosphamide (first course: 0.2 g/day, cumulative total 1 g; second course: 0.4 g). These treatments were given on February 19 and March 15, respectively. During this period, sequential doses of methylprednisolone (80 mg for 3 days and 40 mg for 3 days) were also administered, along with prednisone acetate tablets (40 mg/day), tacrolimus (1 mg, twice daily), tofacitinib citrate tablets (5 mg, twice daily), and pirfenidone maintenance therapy (200 mg, three times daily). On April 5, serum samples were sent to Wuhan Kangsheng Zhenyuan Medical Laboratory Co., Ltd. (Wuhan, China) for a comprehensive dermatomyositis test, which reported anti-Ha antibody IgG level of 287.79 U/ml, anti-MDA5 antibody IgG level of 23.39 U/ml. On April 19, the patient reported weakness in the left lower limb. A complete MRI of both lower limbs revealed soft tissue edema in the upper part of the right calf and the lower part of the left calf, along with a cyst in the right popliteal fossa (**Figure 2**). A follow-up chest CT scan demonstrated a reduction in ground-glass opacities and patchy, linear high-density shadows in both lungs compared to previous findings (**Figure 1F**).Considering the activity of ASS, the patient was discharged on April 22 after completing the third course of methylprednisolone pulse therapy combined with cyclophosphamide treatment. Following discharge, the patient adhered to a regimen of oral prednisone acetate tablets at a dosage of 40 mg/day, tacrolimus at 1 mg twice daily, tofacitinib citrate tablets at 5 mg twice daily, and pirfenidone at 200 mg three times daily. On May 24, the patient self-discontinued the medication due to abdominal pain and dizziness, subsequently experiencing a recurrence of cough, shortness of breath, and difficulty expectorating sputum, accompanied by a fever with a maximum temperature of 38.0°C. The patient was readmitted to the First Affiliated Hospital of Guangxi Medical University on May 30, where hsCRP levels were found to be 39.5 mg/L, and the white blood cell count was 10.75×10^9/L, with a neutrophil percentage of 86.11%. Given the activity of ASS (positive for anti-Ha antibodies) and interstitial pneumonia (possibly viral), the treatment plan included cefoperazone sodium sulbactam sodium at 2g every 12 hours for 12 days, alongside compound sulfamethoxazole tablets at 3 tablets every 6 hours for 4 days for infection control. Additionally, human immunoglobulin was administered at 5g for 3 days for immune regulation, nintedanib at 150 mg for 10 days for anti-pulmonary fibrosis, and prednisone acetate tablets at 20 mg for 8 days, followed by a tapering dose of prednisone acetate tablets at 15 mg for 3 days. Post-treatment, the patient’s body temperature returned to normal, with noted improvements in shortness of breath, cough, and sputum production. A follow-up chest CT on June 6 indicated findings in both lungs (**Figure 1G**).

**Figure 3:**
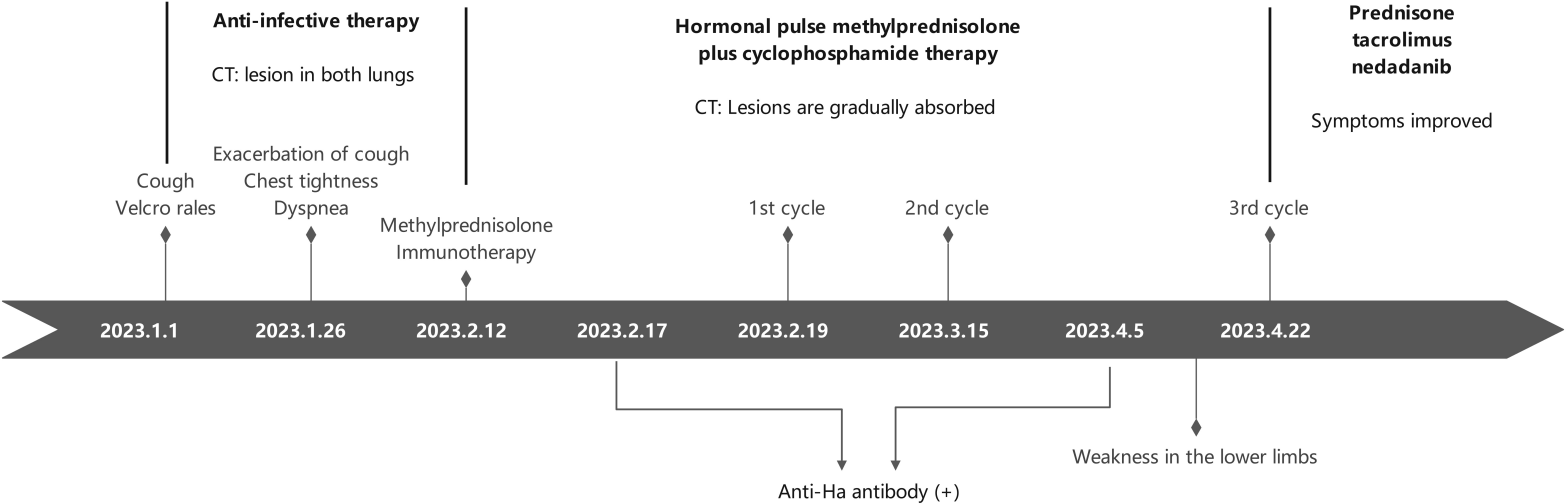
MRI plain scan of the patient’s lower limbs conducted on April 20, 2023, reveals slight swelling in the soft tissues of both the upper and lower regions of the right calf, as well as the lower part of the left calf. The MRI shows a slightly increased T2-weighted imaging (T2WI) signal and blurred interstices.

The confirmed diagnosis was ASS, indicated by the presence of anti-Ha antibodies. Following discharge, the patient continued oral treatment with prednisone, tacrolimus, and nintedanib, with a gradual reduction in dosage. Chest CT scans conducted on July 7 and November 21 revealed a progressive decrease in subpleural patchy, flaky, and linear high-density shadows, as well as ground-glass opacities in both lungs, with no pleural effusion observed on either side (**Figures 1H, I**). Subsequently, prednisone, tacrolimus, and nintedanib were discontinued. As of the latest follow-up, the patient’s condition has remained stable, with no recurrence of respiratory symptoms, fever, or lower limb weakness. The timeline of the patient’s diagnosis and treatment process is presented in (**Figure 3**).

## Discussion

ASS is a type of idiopathic inflammatory myositis (IIM) characterized by the presence of anti-aminoacyl-tRNA synthetase (ARS) antibodies. Clinically, it manifests as interstitial lung disease (ILD), fever, arthritis, mechanic’s hands, and Raynaud’s phenomenon. Although patients with ASS exhibit a specific set of clinical manifestations and possess specific antibodies that aid in diagnosis, these symptoms may present individually or in various combinations and sequences, complicating clinical diagnosis and treatment and increasing the likelihood of misdiagnosis or missed diagnosis. During the onset and progression of ASS, the lungs and muscle tissues are particularly susceptible, resulting in ILD (83%) and myositis (73%). Among these, ILD is the most common extra-muscular clinical manifestation in ASS patients, with an incidence rate ranging from 67% to 100%, significantly higher than that observed in other inflammatory myopathies (1). It is the initial symptom in the majority of ASS patients (2) and serves as the primary cause of morbidity and mortality (3–5). ILD can occur before (9%), after (12%), or simultaneously (40%) with the onset of myositis (6). Therefore, early diagnosis, treatment, and prognosis evaluation of ASS-ILD are crucial in clinical practice.

According to the diagnostic criteria for ASS proposed by Ji et al. in 2010 (7), the presence of anti-aminoacyl-tRNA synthetase (anti-ARS) antibody positivity, along with one or more clinical manifestations, is required for diagnosis. This indicates that anti-ARS antibody positivity plays a crucial role in diagnosing ASS. When the body produces anti-ARS antibodies due to infection or abnormally active autoimmunity, these antibodies target and attack ARS enzymes, promoting the recruitment of antigen-presenting cells and inflammatory cells to the affected organs, which leads to tissue damage. Among these antibodies, anti-Jo-1 is the most common, while rare antibodies such as anti-Ha, anti-KS, and anti-Zo have only been described in case reports (8–11). In the study by Sofia A et al. (12), the positive rates of anti-Ha antibodies in patients with connective tissue disease-related interstitial lung disease (CTD-ILD) and non-CTD-ILD were reported at 3.1% and 1.9%, respectively. However, the prevalence of interstitial lung disease (ILD) in anti-Ha antibody-positive individuals has not been reported. Anti-Ha antibodies are commonly found in myopathies and in 40% of systemic sclerosis cases (13), but they are relatively rare in ASS, leading to frequent misdiagnoses and missed diagnoses in clinical practice. According to statistics, the diagnostic delay for patients with rare types of anti-synthetase antibodies exceeds six months (14). Therefore, when patients present with interstitial lung changes of unknown origin, it is recommended to screen for anti-ARS antibodies to exclude the possibility of ASS.

Currently, there is no clear definition for acute exacerbation of rheumatic disease-associated interstitial lung disease (AE-RD-ILD). It is generally regarded as an acute, clinically significant deterioration in respiratory status, characterized by the emergence of new widespread alveolar abnormalities in patients with a known or concurrent diagnosis of rheumatic disease. The entire duration of the acute exacerbation should be less than one month. Possible triggers typically include infections, as well as non-infectious factors such as the use of DMARDs, surgery, and air pollution. Radiologically, it presents as new ground-glass opacities or consolidations superimposed on pre-existing lesions. It is particularly important to emphasize that comparing imaging findings from each episode with previous imaging and conducting dynamic follow-up are crucial (15).

Through literature searches in multiple databases, including PubMed and Web of Science, this case represents the first reported instance of anti-Ha antibody-positive ASS in China. The patient, an elderly female, initially presented with interstitial pneumonia without other clinical manifestations such as rash, arthritis, or Raynaud’s phenomenon. Initially considered to have community-acquired pneumonia, the patient showed a poor response to anti-infective treatment. However, her respiratory symptoms improved, and the ground-glass opacities in both lungs resolved after the addition of corticosteroids. Upon discontinuation of corticosteroids, the patient experienced an aggravated cough accompanied by fever and fatigue. Follow-up CT revealed rapid progression of interstitial lung changes, and MRI suggested possible myositis in the upper and lower parts of the right calf and the lower part of the left calf. After comprehensive antibody testing excluded connective tissue diseases such as rheumatoid factor (RF), systemic lupus erythematosus (SLE), and vasculitis, idiopathic inflammatory myopathy (IIM) could not be ruled out. Two separate tests for myositis-specific antibodies both indicated positive anti-Ha antibodies, a definitive diagnosis of ASS was established. Moreover, the alleviation of the aforementioned symptoms in the patient following treatment with corticosteroids and immunosuppressants also supports the diagnosis of ASS. Upon reviewing the course of the disease, it was noted that at the onset, the patient exhibited only a significant increase in hsCRP, without an elevation in white blood cell count or neutrophil percentage. The T lymphocyte count and serum ferritin levels were normal. Chest imaging primarily showed interstitial lung changes. Empirical anti-infective treatment was ineffective, but respiratory symptoms improved, and ground-glass opacities decreased after corticosteroids therapy. Therefore, there was insufficient evidence to support a diagnosis of early pulmonary infection or infection combined with AE-RD-ILD in this patient, making the possibility of a non-infectious disease more likely. After discontinuing corticosteroids therapy, the patient’s interstitial lung disease progressed rapidly, accompanied by a decrease in T-lymphocyte count and an increase in serum ferritin levels. It is important to note that pulmonary infections, particularly viral infections, exhibit several clinical manifestations similar to those of CTD-ILD, including cough, dyspnea, fever, decreased T-lymphocyte count, elevated serum ferritin levels, and interstitial lung changes. Furthermore, immune dysfunction or the use of immunosuppressants in patients with connective tissue disease constitutes a significant risk factor for infections. As such, ASS can coexist with infections or other connective tissue diseases, complicating its definitive diagnosis. Therefore, during the diagnostic process in the later stages of this patient’s disease course, it is crucial to seek evidence to ascertain whether the disease progression is solely attributable to active ASS or a combination of ASS and infection, in order to identify the underlying cause and administer targeted treatment. Additionally, hsCRP and serum ferritin may serve as indicators for the progression and prognosis of ASS. Studies conducted by Gono T et al. (16, 17) have demonstrated that patients with elevated ferritin levels exhibit significantly lower survival rates compared to those with lower ferritin levels. For ASS-ILD patients with markedly elevated serum ferritin levels, it is essential to enhance initial treatment and conduct close follow-up observations to improve patient survival rates.

Currently, there is no standardized treatment protocol for ASS. Most cases are empirically treated with corticosteroids and immunosuppressants. Patients with concurrent ILD may also receive antifibrotic treatments such as pirfenidone and nintedanib. Existing case reports and subgroup analyses from cohort studies suggest that pirfenidone may improve lung function in ILD patients (18, 19), potentially enhance survival in patients with clinically amyopathic dermatomyositis accompanied by subacute interstitial pneumonia, and demonstrate favorable tolerability and safety (20). Nintedanib has been shown to exhibit antifibrotic effects in animal models of scleroderma (21). The combination of corticosteroids and immunosuppressants can often reduce the required dosage of corticosteroids. Corticosteroids therapy combined with oral or intravenous cyclophosphamide is utilized for patients experiencing relapses or those unresponsive to glucocorticoid therapy, which can alleviate dyspnea, improve lung function, and enhance chest imaging findings (22). In cases of AE-RD-ILD, given that infections are the primary trigger for most acute exacerbations, it is recommended to discontinue DMARDs treatment and actively implement anti-infective therapy. It is important to note that AE-RD-ILD induced by non-infectious factors is often closely related to the progression of the primary disease. In such instances, intensive and aggressive immunosuppressive therapy is required. However, in actual clinical practice, it is extremely challenging and impractical to completely distinguish between infectious and non-infectious factors. The decision to discontinue or intensify immunosuppressive therapy should be based on individualized treatment according to the patient’s specific condition, as well as common-sense approaches (13). Currently, there is a lack of evidence-based medical research to demonstrate the efficacy of corticosteroids in the treatment of AE-RD-ILD, and the optimal type, dosage, or duration of corticosteroids therapy has yet to be determined.

## Conclusion

In this case, the patient initially presented with cough and interstitial pneumonia, accompanied by a significant increase in hypersensitivity protein at the onset of the disease. No definitive respiratory pathogen was identified, and empirical anti-infective treatment proved ineffective. Symptoms improved, and ground-glass opacities decreased following treatment with methylprednisolone. However, interstitial lung inflammation rapidly progressed after the discontinuation of corticosteroids, accompanied by a decrease in T cell count and an increase in hsCRP and serum ferritin levels, as well as myositis in both lower limbs. Two external tests for dermatomyositis yielded positive anti-Ha antibodies, confirming the diagnosis of ASS and indicating active or progressive ASS, while the possibility of concurrent infection could not be excluded. After three courses of high-dose methylprednisolone pulse therapy and cyclophosphamide immunosuppressive treatment, the patient’s respiratory symptoms significantly improved, and interstitial lung lesions showed absorption compared to previous findings. Once the condition stabilized, corticosteroids were gradually tapered, and treatment was combined with nintedanib and tofacitinib. Follow-up to date has indicated a stable condition with no recurrence, further confirming the diagnosis of ASS and providing valuable insights for related treatments. However, this patient’s case still presents unresolved issues regarding disease progression, such as the potential for pulmonary infection in the early stages and strategies for preventing and differentiating infections during immunosuppressive therapy. Continued follow-up with the patient and in-depth research on the case data and related studies are essential.

## Data Availability

The raw data supporting the conclusions of this article will be made available by the authors, without undue reservation.

## References

[1] Zamora AC Hoskote SS Abascal-Bolado B White D Cox CW Ryu JH . Clinical fea-tures and outcomes of interstitial lung disease in anti-Jo-1 posi-tive antisynthetase syndrome. Respir Med. (2016) 118:39–45. doi: 10.1016/j.rmed.2016.07.009 27578469

[2] Debray MP Borie R Revel MP Naccache JM Khalil A Toper C . Interstitial lung disease in anti-synthetase syndrome: initial and follow-up CT findings. Eur J Radiol. (2015) 84:516–23. doi: 10.1016/j.ejrad.2014.11.026 25541020

[3] Jensen ML Løkke A Hilberg O Hyldgaard C Bendstrup E Tran D . Clinical characteristics and outcome in patients with antisynthetase syndrome associated interstitial lung disease: a retrospective cohort study. Eur Clin Respir J. (2019) 6:1583516. doi: 10.1080/20018525.2019.1583516 30834073 PMC6394310

[4] Marin FL Sampaio HP . Antisynthetase syndrome and autoantibodies: a literature review and report of 4 cases. Am J Case Rep. (2019) 20:1094–103. doi: 10.12659/AJCR.916178 PMC6676984 31344020

[5] Zamarrón⁃de Lucas E Carrera LG Bonilla G Petit D Mangas A Álvarez- Sala R . Antisynthetase syndrome: analysis of 11 cases. Med Clin. (2017) 148:166–9. doi: 10.1016/j.medcli.2016.11.021 28073522

[6] Hervier B Devilliers H Stanciu R Alain M Yurdagul U Agathe M . Hierarchical cluster and survival analyses of antisynthetase syndrome: phenotype and outcome are correlated with anti-tRNA synthetase antibody specificity. Autoimmun Rev. (2012) 12:210–7. doi: 10.1016/j.autrev.2012.06.006 22771754

[7] Ji SY Zeng FQ Guo Q Tan GZ Tang HF Luo YJ . Predictive factors and unfavourable prognostic factors of interstitial lung disease in patients with polymyositis or dermatomyositis: a retrospective study. Chin Med J(Engl). (2010) 123:517–22. doi: 10.3760/cma.j.issn.0366-6999.2010.05.002 20367973

[8] Ghirardello A Doria A . New insights in myositis- peiffi au-toantibodies. Curr Opin Rheumatol. (2018) 30:614–22. doi: 10.1097/B0R00000000000548 30234722

[9] Mahler M Miller FW Fritzler MJ . Idiopathic iffammatory myopathies and the anti-synthetase syndrome: a comprehensive review. Autoimmun Rev. (2014) 13:367–71. doi: 10.1016/j.autrev.2014.01.022 PMC3970575 24424190

[10] Betteridge Z Tansley S Shaddick G Chinoy H Cooper RG New RP . Frequency, mutualexclusivity and clinical associations of myositis autoantibodies in a combined European cohort of idiopathic inflammatory myopathy patients. J Autoimmun. (2019) 101:48–55. doi: 10.1016/j.jaut.2019.04.001 30992170 PMC6580360

[11] Guo Y Wang G Zhou L Wei W . A case of anti-synthetase syndrome with positive anti-Zo antibody. Chin J Rheumatol. (2023) 27:330–2. doi: 10.3760/cma.j.cn141217-20230215-00041

[12] Moll SA Platenburg MGJP Platteel ACM Vorselaars ADM Janssen Bonàs M . Prevalence of novel myositis autoantibodies in a large cohort of patients with interstitial lung disease. J Clin Med. (2020) 9:2944. doi: 10.3390/jcm9092944 32933078 PMC7563342

[13] Tansley SL Betteridge Z Lu H Davies E Rothwell E New PP . The myositis clinical phenotype associated with anti-Zo autoantibodies:A case series of nine UK patients. Rheumatology. (2019) 59:1626–31. doi: 10.1093/rheumatology/kez504 PMC7310094 31665469

[14] Cavagna L Trallero-Araguás E Meloni F Cavazzana I Rojas-Serrano J Feist E . Influence of antisynthetase antibodies specificities on antisynthetase syndrome clinical spectrum time course. J Clin Med. (2019) 8:2013. doi: 10.3390/jcm8112013 31752231 PMC6912490

[15] Luppi F Sebastiani M Salvarani C Bendstrup E Manfredi A . Acute exacerbation of interstitial lung disease associated with rheumatic disease. Nat Rev Rheumatol. (2022) 18:85–96. doi: 10.1038/s41584-021-00721-z 34876670

[16] Gono T Kawaguchi Y Hara M Masuda I Katsumata Y Shinozaki M . Increased ferritin predicts development and severity of acute interstitial lung disease as a complication of dermatomyositis. Rheumatology. (2010) 49:1354–60. doi: 10.1093/rheumatology/keq073 20385617

[17] Zhao N Jiang W Wu H Wang P Wang X Bai Y . Clinical features, prognostic factors, and survival of patients with antisynthetase syndrome and interstitial lung disease. Front Immunol. (2022) 13:872615. doi: 10.3389/fimmu.2022.872615 36032132 PMC9399497

[18] Miura Y Saito T Fujita K Tsunoda Y Tanaka T Takoi H . Clinical experience withpirfenidoneinfivepatientswithscleroderma-relatedinterstitial lung disease. Sarcoidosis Vasc Diffuse Lung Dis. (2014) 31:235–8. 25363224

[19] Khanna D Albera C Fischer A Khalidi N Raghu G Chung L . An open-label, phase IIstudy of the safety and tolerability of pirfenidone in patientswith scleroderma -associated interstitial lung disease: theLOTUSS trial. J Rheumatol. (2016) 43:1672–9. doi: 10.3899/jrheum.151322 27370878

[20] Li T Guo L Chen Z Gu L Sun F Tan X . Pirfenidone in patients with rapidlyprogressive interstitial lung disease assocated with cinicallyamyopathic dermatomyositis. Sci Rrp. (2016) 6:33226. doi: 10.1038/srep33226 PMC5018967 27615411

[21] Group of Pulmonary Vascular and Interstitial Diseases Associated with Rheumatic Diseases, Chinese Association of Rheumatology and Immunology Physicians; Chinese Rheumatic Disease Data Center . 2018 Chinese expert-based consensus statement regarding the diagnosis and treatment of interstitial lung disease associated with connective tissue diseases. Zhonghua Nei Ke Za Zhi. (2018) 57:558–65. doi: 10.3760/cma.j.issn.0578-1426.2018.08.005 30060326

[22] Connors GR Christopher-Stine L Oddis CV Danoff SK . Interstitial lung disease associated with the idiopathic inflammatory myopathies:what progress has been made in the past 35 years? Chest. (2010) 138:1464–74. doi: 10.1378/chest.10-0180 21138882

