# Correction: Acute exacerbation of anti-Ha antibody-positive antisynthetase syndrome-associated interstitial lung disease: a case report

**DOI:** 10.3389/fimmu.2025.1647113

**Published:** 2025-06-26

**Authors:** Yinying Li, Junyi Ke, Shu Huang, Shiya Lin, Siyu Lei, Hongchun Huang, Luxi Lei, Qinglan Li, Yuanlan Wu, Minchao Duan

**Affiliations:** ^1^ Guangxi Medical University, Nanning, Guangxi, China; ^2^ Department of Respiratory and Critical Care Medicine, Wuming Hospital of Guangxi Medical University, Nanning, Guangxi, China

**Keywords:** anti-synthetase syndrome (ASS), anti-Ha antibody, ILD, case report, corticosteroids therapy

In the published article, there was an error in affiliation 1. Instead of “The Second Clinical Medical College, Guangxi Medical University, Nanning, Guangxi, China”, it should be “Guangxi Medical University, Nanning, Guangxi, China”.

The authors apologize for this error and state that this does not change the scientific conclusions of the article in any way. The original article has been updated.

